# Contribution of Entrepreneurship to the Social Integration of People Intellectual Disabilities: A Case Study Based on the Analysis of Social Networks

**DOI:** 10.3389/fpsyg.2021.725060

**Published:** 2021-10-12

**Authors:** Virginia Barba-Sánchez, Yolanda Salinero, Pedro Jiménez-Estevez, Esteban Galindo

**Affiliations:** ^1^Business Administration Department, ESII, Universidad de Castilla-La Mancha, Albacete, Spain; ^2^Business Administration Department, Faculty of Legal and Social Science, Universidad de Castilla-La Mancha, Toledo, Spain; ^3^CECAP, Ronda Buenavista, Toledo, Spain

**Keywords:** entrepreneurship, intellectual disabilities, social integration, social networks, case study

## Abstract

In an environment characterized by high unemployment rates among people with disabilities, the objective of the present work is to analyze entrepreneurship as a labor option which fully inserts people with intellectual disabilities (PwID) into their societies. In order to carry out this research, a case study methodology based on social network analysis has been adopted, given the nature of the variables analyzed. The results indicate that the fact of having managed to start up the company has been an important source of self-confidence and inspiration, as well as increasing and intensifying the social networks of PwID involved in the entrepreneurial project.

## Introduction

In an environment characterized by high unemployment rates among people with disabilities (PwD), this group’s social incorporation is undermined by their economic dependence both on their families and on public institutions (ODISMET, 2018). According to data from the INE (2020), in 2019, the unemployment rate among PwD in Spain exceeded that of the non-disabled population by more than ten points (23.9% compared to 17.1), a gap that has consisted during the evolution of such data since records have been kept. Although the main objective of Law 13/1982 (Law on the Social Integration of the Disabled) has been to reduce this gap at a national level in order to achieve the full social integration of this group, we observe that, in fact, this has not been the case (Calderón-Milán et al., 2020); the relevance of this problem is underlined by the fact that, at the national level, almost two million people of working age (from 16 to 64years of age) have some form of disability, either permanent or temporary, thereby representing 6.2% of the total working-age population (INE, 2018). At the level of the European Union (EU), this percentage is approximately 16%, a percentage which is likely to increase as the population ages (Halabisky, 2014).

Manzanera-Román and Brändle (2019) point to employment as one of the most effective elements in the integration of people into society. Authors such as Bengisu and Balta (2011) suggest that the use of PwD would improve the quality of service and efficiency, reducing the employing company’s costs. In practice, however, discrimination by employers plays a major part in PwD’s low employment rates (Hashim and Wok, 2014; Beisland et al., 2016), while those who have been employed tend to be employed in low-skill and low-paid occupations (Meager and Higgins, 2011). In short, the problem is not only that PwDs have a high unemployment rate, but that those who do get a job often find it unsatisfactory (Darcy et al., 2020).

In this context, self-employment is a useful alternative (Dhar and Farzana, 2017; Barba-Sánchez et al., 2019), which contributes to PwD’s social inclusion (Maritz and Laferriere, 2016) and which, even in a worst-case scenario, serves as a training program to improve employment (Doyel, 2002: Bruce and Schuetze, 2004). Furthermore, authors such as Pagán (2009) and Halabisky (2014) consider this alternative appropriate for PwD given that it can give them higher work flexibility in terms of workload and working hours, thereby allowing a better balance between their disability and their working life. Akinyemi (2016) points out that self-employment gives PwD the opportunity to be productive members of society, which, according to authors such as O’Neil et al. (2020) or Seelman (2008), increases their self-esteem and self-confidence. However, inclusive entrepreneurship defined by Maritz and Laferriere (2016, p. 46), as that which “contributes to social inclusion to give all people an equal opportunity to start and operate a business” in general, and more specifically inclusive entrepreneurship developed by PwD is a subject that is scarcely addressed both in specialized literature (Renko et al., 2015; Bagheri and Abbariki, 2017) and in public policies that promote it (Muñoz et al., 2019).

Among PwD, PwIDs are particularly vulnerable as they “tend to be largely sedentary and passive with negative implications for their self-confidence, constructive engagement time, environmental sensory input, and social status” (Lancioni et al., 2017 p. 282). However, previous literature on entrepreneurship among this group of PwID in the growing body of empirical research on entrepreneurs with disabilities (EwD) is almost non-existent (Hutchinson et al., 2021). This lack of knowledge presents policy makers and practitioners involved in the promotion and development of entrepreneurship among PwID with a challenge in developing effective strategies and programs (Bagheri and Abbariki, 2017). Hence, the objective of this work is to contribute to the study of entrepreneurship among PwID, specifically investigating the question: does entrepreneurship favor the social integration of PwID?

## Entrepreneurship, Social Networks Theory and Social Integration

Keyes (1998 p. 122) defines social integration as “the evaluation of the quality of the relationships we maintain with society and the community.” Following the Social and Cultural School of Thought (Dhar and Farzana, 2017), most of the time, entrepreneurship is undertaken when people feel supported by their social and cultural environment. From such thinking, the “Social-Inclusive” Model is born, which interprets the reality of PwID from the guarantee of fundamental rights. Thusly, it is assumed that it is the person themselves who should be the active participant in achieving their personal objectives, while the family, in turn, should serve as a support tool for achieving those objectives, maintaining an active role that facilitates access to the framework of fundamental rights; society, therefore, must be permeable, understanding PwID as another member of society, with all the rights and duties that pertain to such membership.

However, the disabilities not only affect individuals but also family groups, the local social environment and economy, society in general and its pertinent public authorities (Ortíz-García and Olaz-Capitán, 2021), this type of inclusive entrepreneurship cannot be analyzed solely from the perspective of Stakeholder’s Theory (Barba-Sánchez et al., 2021).

Social network theory allows the analysis of egocentric or personal networks, which are those centered on specific individuals and on which the studies of their behavior are focused, since they show the relationships of the context in which these individuals are immersed or anchored (Scott, 1991). Thus, social networks are a form of representation in which an attempt is made to identify the various social structures of human relationships (Barba-Sánchez and Molina-Ramírez, 2015). Lee et al. (2005) classify them as close primary networks, made up of members of the nuclear family, non-close primary networks, made up of friends, neighbors and work colleagues, and secondary networks, which include the rest of professional contacts.

Among the main conclusions of the Report on the Disability Entrepreneurship Ecosystem in Australia (Darcy et al., 2020), is the importance of social networking, not only for the success of the business project, but also for the personal development and social integration of PwIDs. The creation of the company favors the increase in the PwID’s professional networks. Furthermore, having a larger network of contacts contributes both to job placement and mobility (Győri et al., 2019) and to alleviating and preventing social isolation (Thoresen et al., 2018). However, PwID often have much smaller social networks than people with other disabilities (van Asselt-Goverts et al., 2014).

Given that the aim of the present work was to analyze entrepreneurship as a labor option which fully inserts PwID into their societies, we intend to visualize the evolution of the entrepreneurial team’s social networks, classifying them into personal networks, such as family and friends, and professional networks, such as customers, suppliers, and public institutions.

## Methodology

In order to carry out this research, we have carried out a qualitative study that can be assimilated to a case study in which the information collected was done through in-depth interviews, which are a valid method for collecting information in numerous empirical and qualitative studies. However, due to the exploratory nature of this research and space limitations, we have only included the data in the graph that summarize the information collected. A social network analysis (SNA) has been adopted, given the nature of the variables analyzed. Case studies allow an analysis of the phenomenon under investigation within its real context, using multiple sources of evidence, both quantitative and qualitative (Villarreal and Landeta, 2010). In Yin’s words (1989) when he speaks of the definition of a case study, he points out that they are empirical investigations which study a contemporary phenomenon within its real context, where the limit between the phenomenon and the context are not precisely shown, and in which multiple sources of evidence are used. For its part, SNA is based on social network theory, whose main axiom is “that a node’s position in a network determines in part the opportunity and constraints that it encounters, and in this way plays an important role in a node’s outcomes” (Borgatti et al., 2009 p. 894). In this sense, according to Rutte et al. (2010), SNA allows us to understand the spatial dimension of the social capital of individuals. Specifically, the current study’s methodology consists of drawing the relational social networks of PwID before and after undertaking entrepreneurship (the so-called ego network), observing the differences between the two temporal moments.

This technique not only allows us to draw the existing relationship for each actor (vertexes) but also the intensity of the relationship between two actors (edge thickness) and the direction of that relationship (arcs). In our case, the intensity of the relationship has been measured on a five-point Likert scale where 1 was an unknown person; 2, a known person; 3, a known person with a weak connection and sporadic interactions; 4, a known person with medium connection and frequent interactions; and 5, a known person with strong connection who is part of the entrepreneur’s daily life.

Our current analysis focused on a case of inclusive and collective entrepreneurship carried out entirely by PwID: coffee fertilizer. This cooperative company arose as a result of the Entrepreneurship Course for PwID taught by the University of Castilla-La Mancha (UCLM) during the 2016/17 Academic Year, and under the supervision and support of the Social Business Factory (SBF), a business incubator and seedbed for creating, monitoring, and advising of socially inclusive companies. Created in 2018 by 14 partners, this cooperative depends on a circular economic model that elaborates ecological hummus (fertilizer) from the waste of coffee grounds using the Californiana red worm. After its creation, 8 more partners joined the project, bringing the current number of partners to 22 (see Table 1 with description of the 22 Cooperative members). Table 2 lists all the stakeholders with whom at least one of the members of this cooperative has a relationship before and/or after the creation of the company.

**Table 1:** Cooperative members detail of Abono Café.

Cooperative member	Gender	Age	Type of disability	Degree of disability	Educational level
CM1	Female	31	Psychica	65%	School Graduate
CM2	Female	26	Psychica	41%	Secondary level education
CM3	Female	31	Psychica	65%	Secondary level education
CM4	Female	35	Psychica	65%	Secondary level education
CM5	Female	33	Psychica	65%	Secondary level education
CM6	Female	28	Psychica	65%	Secondary level education
CM7	Male	41	Psychica	68%	School Graduate
CM8	Female	22	Psychica	81%	Secondary level education
CM9	Female	35	Psychica	65%	Primary School
CM10	Male	33	Psychica	65%	Basic Professional Training
CM11	Hombre	38	Psychica	65%	No schooling completed
CM12	Male	47	Psychica	65%	No schooling completed
CM13	Female	43	Psychica	65%	School Graduate
CM14	Hombre	26	Psychica	70%	Secondary level education
CM15	Male	27	Psychical, physical, and sensory	76%	Primary School
CM16	Female	31	Psychica	65%	Primary School
CM17	Female	31	Psychica	69%	School Graduate
CM18	Female	25	Psychica	65%	School Graduate
CM19	Female	29	Psychica	67%	Initial Professional Qualification Program
CM20	Male	31	Psychica	65%	Primary School
CM21	Female	26	Psychica	65%	Primary School
CM22	Male	30	Psychica	65%	Primary School

**Table 2:** Stakeholders participating in the study.

Stakeholder ID	Description
CoopMember#	Cooperative Member Number #. It is necessary to distinguish the first fourteen from the following eight, who joined the project after its creation.
PresidentCoop	President of the Cooperative and member #3.
SecretaryCoop	Secretary of the Cooperative and member #11.
TreasureCoop	Treasurer of the Cooperative and member #13.
ProfessionalCounsellor	Professional Couch that accompanies participants during entrepreneurial process.
CECAP	Staff of the social entity CECAP, a Training Service based in Toledo who collaborated with the project.
SBF	Staff of the Social Business Factory group, support platform, and monitoring of the entrepreneurial process.
UCLMteacher	Professors of the course Entrepreneurship and Specificity, articulated through the UCLM.
EOIteacher	Professors of the entrepreneurship course organized by the School of Industrial Organization (EOI).
EOIstaff	Management team of the entrepreneurship course organized by EOI.
TownHall	Members of Toledo City Hall sympathizing with the project.
JCCM	Members of Regional Government of Castilla-La Mancha sympathizing with the project.
CigarralBusiness	Management team of the *Cigarral del Ángel Custodio*. First business partner with whom an agreement was signed for the temporary transfer of space; the EU committee was received at its facilities and came to visit the project.
GrecoMuseumStaff	Management team of the Greco Museum. First client of the company.
Children&Families	Children and family members attending the awareness-raising workshop organized by the Greco Museum to promote the entrepreneurial project.
Caixabank	Director of the Caixabank office where the company’s account was made.
CaixabankVolunteers	Team of Caixabank volunteers, who offered a financial economy workshop to the entrepreneurs.
Neighbours	Group of gardening neighbors who manage an urban gardening project and share part of the premises where the company is located.
CafeNetwork	Network of cafeterias that collaborate with the project.
BioenergyBusinesses	Entrepreneurs of the bioenergy company with which negotiations are being established.

## Results and Discussion

### Overview of the Interview Results

Upon analyzing the case study, we can summarize the following results:

First of all, participants’ families had a high level of involvement in the early stages of the project, helping to establish the cooperative’s infrastructure. There were many interactions between families, which extended to the relationships between the participants themselves. At some point, this interaction damaged the relationships between some families and strengthened trust between others. The relationship of the participants with their own families did not change due to the entrepreneurship process *per se*, although it is true that the context generated with the business project has facilitated the professional team’s training efforts in this sense, working transversally with other areas of the entity. It would be complicated to establish the relational flows in this aspect or to try to measure its average.

“One of the cooperative members, as a result of working in the cooperative, went to live in a sheltered flat, his mother said that before this he would never have imagined that he would have had the self-confidence to take this step.”

Relationship between equals. All entrepreneurial processes promote collaboration and teamwork and the technical counseling that participants received placed great importance on cultivating these aspects in order to produce a cohesive group. At the quantitative level, the evolution that participants had regarding interpersonal and group relations based on the identity of the entrepreneur/worker is interesting.

- Participants. Related to the previous point, it is important to underline how each participant evolved parallelly in terms of maturation, self-concept and self-knowledge. In this way, the relations toward their equals and toward themselves undergo constant translation with a positive tendency toward personal and interpersonal evolution. As in the case of the families, it would be complicated to establish relational flows in the aspect or to try to measure their mean.

**Figure 1:**
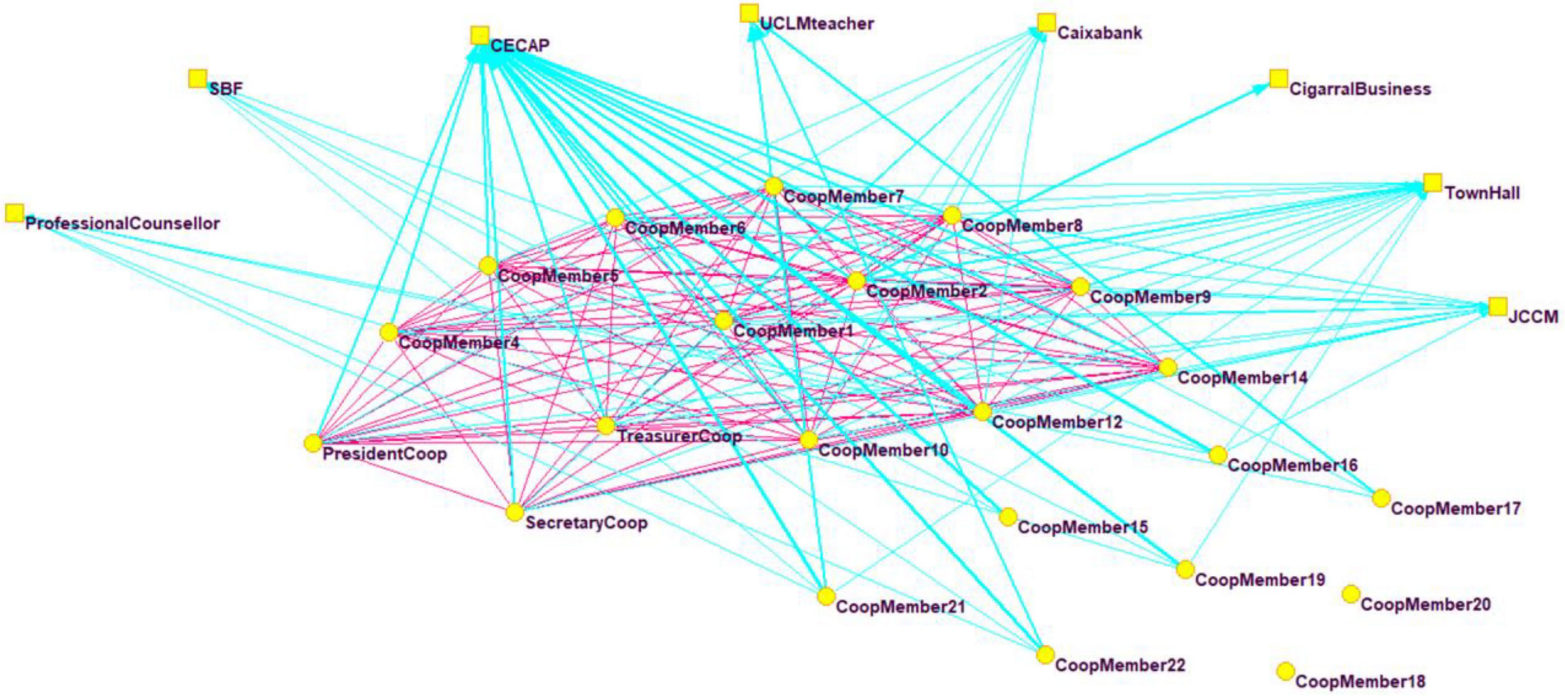
Network of entrepreneurs before company creation. The members/workers of the cooperative (internal stakeholders) are represented by circles, and their relationships are displayed in fuschia, while external stakeholders (organizations that maintained some kind of contact prior to the creation of the company) are represented by squares, and their relationships are drawn in blue.

With regard to the SNA, as can be observed in Figure 1, the main link between all future entrepreneurs, prior to the creation of the company, was CECAP. In addition, the relations between the entrepreneurial team’s members (drawn in fuschia) were weak (among the fourteen initial promoters) and even, in some cases, non-existent (among the eight new members).

**Figure 2:**
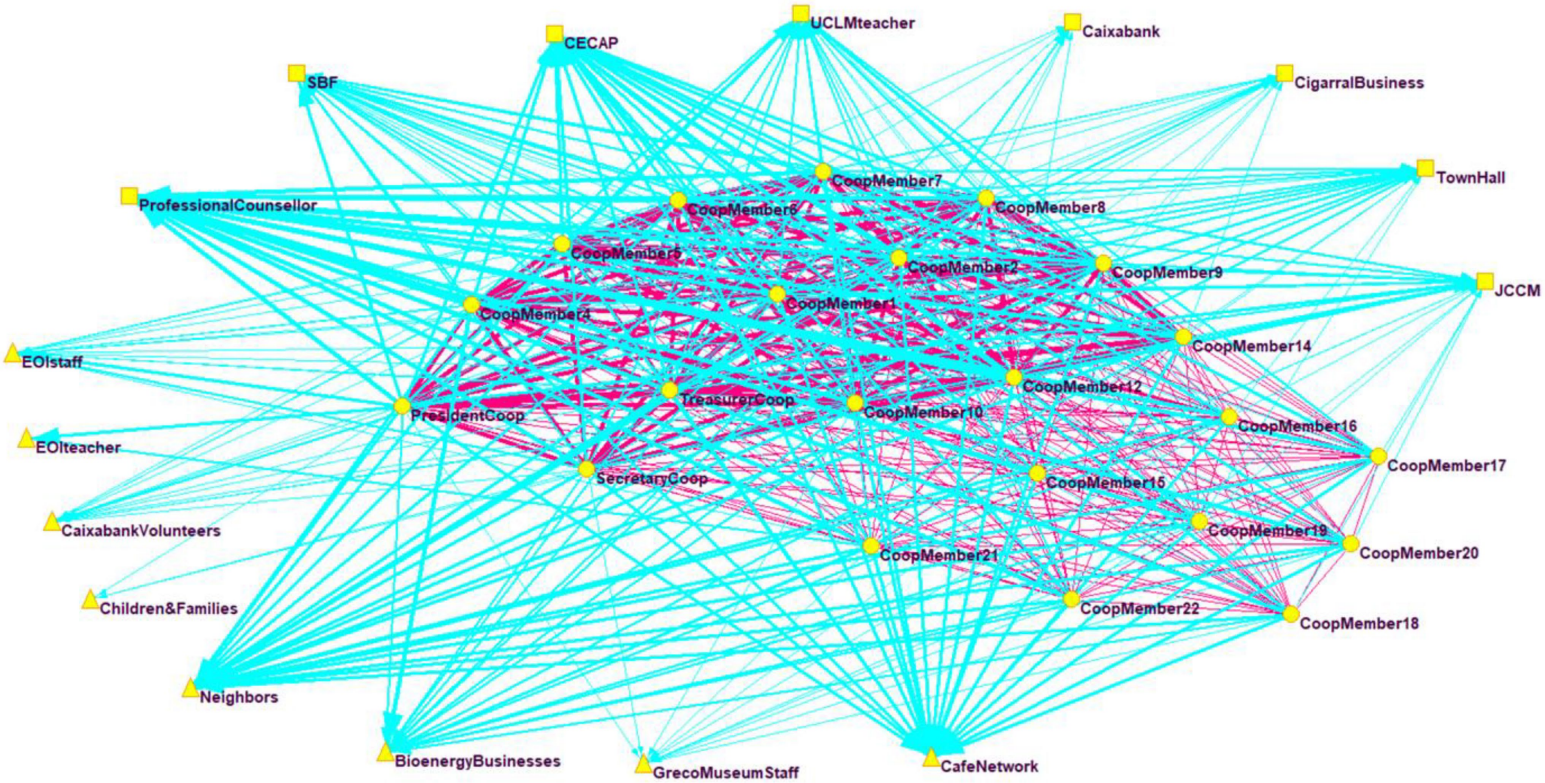
Network of entrepreneurs after company creation. The members/workers of the cooperative (internal stakeholders) are represented by circles, while external stakeholders (organizations that maintained some kind of contact prior to the creation of the company) are represented by squares and triangles (new contacts). The relations between the members of the company are drawn in fuschia and the external ones in blue.

Once the company had been established, the generation of an ego network (Figure 2) is observed in a dense network among the members of the company (especially among the initial promoters of the company), which is represented by the fuschia area. Additionally, their relations with external stakeholders have multiplied (eight new stakeholders were added, represented by triangles) and intensified considerably (greater edge thickness), especially the relations with the professional guide, with the network of cafés that supplied the raw material and with the neighboring orchards that share part of the municipally owned campus where the company is located.

## Conclusion

The results indicate that the fact of having managed to start up the company has been an important source of self-confidence, in line with what Tirado-Valencia et al. (2021) say as well as increasing and intensifying the social networks of PwID involved in the entrepreneurial project, in line with results obtained by previous literature (Conroy et al., 2010; Thoresen et al., 2018; Caldwell et al., 2020). However, the process has faced additional challenges, such as social reticence and having to overcome the limitations derived from disability, which are conclusions of interest for policy makers and other social agents involved in disability.

During the entire process of training, creating the company and carrying out the entrepreneurial activity, an essential and fundamental pillar has been the support and interest of the students’ families: They are not mere spectators of the project, but also implicate themselves, helping students in some activities (a fact that strengthens family union even more, if possible). Additionally, it has been indispensable that the students have had the collaboration of a member of the social entity CECAP, who assigns each cooperative member the task that is most adequate for him/her and in which they can best develop their capacities.

One of the limitations of our study is that we have only collected evidence from one company, the first that has emerged from the inclusive entrepreneurship courses. It is foreseen that this line of work will continue at the University of Castilla-La Mancha (UCLM); in other words, we intend to continue giving courses and to continue supporting entrepreneurial ideas, with the hopes of expanding our sample with more companies created by PwID in the near future. As in Europe, the United States, and Australia (Larsson, 2020), there are no data on the number of Spanish companies owned by PwID (Ortíz-García and Olaz-Capitán, 2021), so a relevant future work would be to quantify the group of entrepreneurs with intellectual disabilities (EwID).

Our work is eminently descriptive because what we are looking for is a study of the relationships between entrepreneurs and their networks. As future lines of inquiry, we will analyze, following the evidence pointed out by Hutchinson et al. (2021), whether these new relationships provide these entrepreneurs with a better quality of life and greater integration within their social environment.

## Data Availability Statement

The original contributions presented in the study are included in the article/supplementary material, further inquiries can be directed to the corresponding author.

## Author Contributions

VB-S, PJ-E, and YS have made the theoretical review and empirical study. EG has developed the interviews. All authors contributed to the article and approved the submitted version.

## Funding

This work was supported by the Faculty of Legal and Social Science, University of Castilla-La Mancha, 45071 Toledo, Spain.

## Conflict of Interest

The authors declare that the research was conducted in the absence of any commercial or financial relationships that could be construed as a potential conflict of interest.

## Publisher’s Note

All claims expressed in this article are solely those of the authors and do not necessarily represent those of their affiliated organizations, or those of the publisher, the editors and the reviewers. Any product that may be evaluated in this article, or claim that may be made by its manufacturer, is not guaranteed or endorsed by the publisher.
